# NK Cell Subsets Changes in Partial Remission and Early Stages of Pediatric Type 1 Diabetes

**DOI:** 10.3389/fimmu.2020.611522

**Published:** 2021-01-25

**Authors:** Laia Gomez-Muñoz, David Perna-Barrull, Adrian Villalba, Silvia Rodriguez-Fernandez, Rosa-Maria Ampudia, Aina Teniente-Serra, Federico Vazquez, Marta Murillo, Jacobo Perez, Raquel Corripio, Joan Bel, Marta Vives-Pi

**Affiliations:** ^1^ Immunology Service, Germans Trias i Pujol Research Institute, Autonomous University of Barcelona, Badalona, Spain; ^2^ Endocrinology Service, Germans Trias i Pujol University Hospital, Badalona, Spain; ^3^ Pediatrics Service, Germans Trias i Pujol University Hospital, Badalona, Spain; ^4^ Department of Pediatric Endocrine, Parc Tauli Hospital Universitari, Institut d’Investigació i Innovació Parc Taulí I3PT, Universitat Autonoma de Barcelona, Sabadell, Spain

**Keywords:** NK cell subpopulations, type 1 diabetes, partial remission, pediatrics, autoimmunity

## Abstract

Type 1 diabetes (T1D) is a chronic metabolic disease characterized by the autoimmune destruction of β-cells in the pancreatic islets. T1D is preceded by islet-specific inflammation led by several immune cells. Among them, natural killer (NK) cells are emerging as important players in T1D development. Human NK cells are characterized by CD56 and CD16 expression, which allows classifying NK cells into four subsets: 1) CD56^dim^CD16^+^ or effector NK cells (NK_eff_); 2) CD56^bright^CD16^−^ or regulatory NK cells (NK_reg_); 3) intermediate CD56^bright^CD16^+^ NK cells; and 4) CD56^dim^CD16^−^ NK cells, whose function is not well determined. Since many studies have shown that T1D progression is associated with changes in various immune cell types, we hypothesize that the kinetics of NK cell subsets in the blood could correlate with different stages of T1D. To that aim, pediatric patients newly diagnosed with T1D were recruited, and peripheral NK cell subsets were analyzed by flow cytometry at several disease checkpoints: disease onset, partial remission (PR), 8 months (for non-remitters), and 12 months of progression. Our results showed that total NK cells and their four subsets are altered at the early stages of T1D. A decrease in the counts and percentage of total NK cells and NK_eff_ cells at the different disease stages was found when compared to controls. These results suggest the extravasation of these cells into the islets at disease onset, which is maintained throughout the follow-up. By contrast, NK_reg_ cells increased during the early stages after T1D onset, and both intermediate NK cells and CD56^dim^CD16^-^ NK cells diminished at the PR stage, which might reflect the immunoregulatory attempts and could be candidate biomarkers for this stage. Also, CD56^dim^CD16^-^ NK cells increased during T1D progression. Finally, changes in CD16 expression were identified in the different T1D stages, highlighting a CD16 expression reduction in total NK cells and NK_eff_ cells 1 year after diagnosis. That may reflect a state of exhaustion after multiple cell-to-cell interactions. Altogether, our preliminary data provide a longitudinal picture of peripheral NK cell subpopulations during the different T1D stages, which could be potential candidate biomarkers indicators of disease progression.

## Introduction

Type 1 diabetes (T1D) is an autoimmune disease caused by the destruction of the insulin-producing β-cells. T1D most often arises in children, and after diagnosis, up to 80% of pediatric patients experience a spontaneous and transient partial remission (PR) phase, also known as honeymoon, which can last up to 2 years (1). This interesting period is defined by low requirements of exogenous insulin and diminished glycated hemoglobin (HbA1c) (2, 3), and could be the result of metabolic and immunological interactions that allow the recovery of β-cell function. Because early stages of T1D are often chosen for immune interventions (4), to characterize new biomarkers and cell subset changes at this time-point is essential.

Autoreactive T cells are key mediators of β-cell destruction, but other leukocytes—macrophages, dendritic cells, natural killer (NK) cells, and B lymphocytes—are determinant in the immunopathogenesis of the disease, especially at the initial stages of the autoimmune attack. Among the innate immune cells, NK cells can participate in the initiation and maintenance of autoimmune responses (5–7). These cells modulate several innate and adaptive components by their cytotoxic activity, which is regulated through a balance of activator and inhibitory receptors, and by the production of cytokines and chemokines (8). In T1D, NK cells infiltrate the islets, being one of the first immune cells to extravasate into the pancreas, and can be cytolytic for β-cells. Studies in experimental models support an active role of NK cells in T1D pathogenesis (9–11), and most data point to NK contribution to the aggressivity of insulitis and the acceleration of the disease (12). However, their role in human T1D is controversial, with both protective and destructive functions (13, 14). This fact could be explained by the different functions of NK cell subsets.

In human peripheral blood, NK cells are defined by the lack of CD3 and the expression of the cell adhesion molecule CD56. Moreover, based on the cell surface density of CD56 and the expression of CD16 (also known as FcγRIII), NK cells can be subdivided into four different subsets (15, 16). Comprising around 90% of circulating NK cells, CD56^dim^CD16^+^ or effector NK (NK_eff_) cells possess a potent cytotoxic activity, while the remaining 10% characterized by CD56^bright^CD16^-^ or regulatory NK (NK_reg_) cells, mainly produce and secrete diverse cytokines—including the immunoregulatory ones. The other two subsets are CD56^bri^CD16^+^ or intermediate NK cells, which represent a functional intermediate stage of maturation between regulatory and effector NK cells (17), and CD56^dim^CD16^−^ NK cells, a unique non-classical subset that is not fully characterized.

Abnormalities in peripheral blood NK cell frequency and activity have been described in patients with T1D (18, 19). The reduced frequencies of NK cells found in recently diagnosed patients with T1D (20–22) probably correlate with an increase of these cells in the insulitis and pancreatic lymph nodes, thus reflecting the chronic aggression. However, the analysis of different NK cell subsets or their activation status could contribute to the elucidation of the exact role of NK cells in different stages of T1D, including the PR phase. In this regard, CD16 is a molecule found on the membrane of NK cells, especially in all CD56^dim^ NK cells, which is involved in antibody-dependent cell cytotoxicity (ADCC), triggering NK degranulation and the killing of target cells (23). Activated NK cells lose CD16 expression on the membrane, probably to modulate NK cell function (24).

Since many studies have shown that the progression of T1D is associated with changes in various immune cell types and little is known about the contribution of the NK cell subsets in the different T1D stages, this pilot study is aimed at analyzing the frequency and absolute counts of these cells in peripheral blood at key time-points and to assess their CD16 expression in order to characterize their effector cytolytic activity.

## Materials and Methods

### Study Population

Pediatric patients with T1D at disease onset (n = 17) and age- and sex-matched non-diabetic control subjects (n = 17) were included in this study. All patients fulfilled the American Diabetes Association classification criteria for T1D (25). Inclusion criteria were 4–18 years of age and normal body mass index (BMI) according to the Spanish BMI pediatric cohort growth chart (26). Exclusion criteria were being under immunosuppressive or anti-inflammatory treatment, the presence of other autoimmune diseases, type 2 diabetes, pregnancy, compromised kidney function, or liver diseases. All the experiments were carried out in strict accordance with the principles outlined in the Declaration of Helsinki for human research and after the approval of the Committee on the Ethics of Research of the Germans Trias i Pujol Hospital and Hospital Parc Taulí. Legal representatives of all participants signed informed consent forms at the beginning of the study.

### Study Design

Blood samples were obtained at three different time-points through T1D progression: at disease onset (n = 17), at PR (n = 11) or 8 months (8M) for non-remitter patients (n = 6), and 12 months (12M) after disease onset (n = 6). At disease onset, samples were collected between 1 and 14 days after diagnosis. At this stage, all patients with T1D were tested for autoantibodies to GAD65, IA-2, and ZnT8. Patients were considered to be in PR when they fulfilled the accepted criteria of ≤9 insulin dose-adjusted HbA1c (IDAA1c), an index that is calculated as HbA1c (%) + [4 × insulin dose (U/kg/day)] (3). Longitudinal data collection occurred over 1 year. So far, only six patients have completed the year of follow-up since diagnosis and were included in this study.

### Sample Collection

Blood samples of 6 ml from T1D patients were collected at three longitudinal time-points in EDTA tubes (BD Biosciences, San Jose, CA, USA) and processed within 6 h. Control samples from non-diabetic subjects (sex and age-related) were obtained simultaneously to the diagnosis of children with T1D.

### Metabolic Parameters

HbA1c was determined by high-performance liquid chromatography (ADAMS A1c HA-8180V, Arkray, MN, USA) in all patients at each time-point. Basal non-fasting C-peptide was determined by ELISA (Architect i2000, Abbott, IL, USA) in both controls and patients at each time-point.

### HLA Class II Typing

HLA typing of DRB1 alleles was determined in all patients at the Immunology Service by hybridization with sequence-specific oligonucleotide (SSO) probes (LABType SSO, One Lambda, CA, USA), following the manufacturer’s instructions. According to HLA-DRB1 allele combinations, three groups of HLA-DRB1 genotypes were formed: high-risk (positive for both HLA-DRB1*03 and HLA-DRB1*04 or homozygous for these alleles), moderate risk (positive for HLA-DRB1*03 or HLA-DRB1*04), and neutral risk (negative for HLA-DRB1*03 and HLA-DRB1*04).

### Flow Cytometry

Phenotypic analysis and the median fluorescence intensity (MFI) of CD16 of NK cell subpopulations in fresh peripheral blood were performed through multicolor flow cytometry with a four-laser LSR Fortessa cytometer (BD Biosciences). For flow cytometry assays, quality control procedures were implemented weekly. Briefly, 1–2 ml of peripheral whole blood were washed with 15 ml of FACSFlow Sheath Fluid (ThermoFisher Scientific), and 100 μl were incubated with the corresponding monoclonal antibodies. For NK phenotyping, the panel of antibodies was built as follows (27): CD45 AF700 (clone HI30, BioLegend), CD3 APCH7 (clone SK7, BD Biosciences), CD19 APCH7 (clone HIB19, BD Pharmingen), CD14 V450 (clone MφP9, BD Horizon), CD16 APC (clone B73.1, BD Pharmingen), and CD56 PE (clone MY31, BD Biosciences). After incubating the cells for 20 min, erythrocytes were lysed for 7 min (Lysing Buffer, BD Biosciences). Absolute counts (cells/μl) were analyzed for all subsets using Perfect Count Microspheres of known concentration (Cytognos SL, Salamanca, Spain). At least 10.000 leukocyte events per sample and 5.000 beads were acquired using LSR Fortessa Flow Cytometer (BD Biosciences). The gating strategy to analyze specific NK cell subsets was based on international consensus (28). Data were analyzed using FACSDiva software (BD Biosciences). The CD56^bri^CD16^-^ population was used as an internal control population in the analysis of the MFI of CD16.

### Statistical Analysis

Cell concentration, percentage, and MFI data were imported into R with the pzfx R package. Data reformatting, filtering, and exploration were assessed with tidyverse and ggplot2 suites of packages. A generalized linear mixed model was fit with condition, time or time × remission as fixed effects and sample identifier and individual as random effect covariates using the *glmer* function in the lme4 R package (29), and the proportion of cell counts in the cluster of interest relative to total input cell population as response variable following a binomial distribution as implemented in the CyTOF workflow (30). Sex and age were excluded from the final model due to a lack of significant effects. Contrast p-values were generated with the NK fraction. Values were analyzed referred to either total NK cell counts or PBMC input cell counts. Differences in ratios between CD56^dim^CD16^−^ and NK_reg_ relative to NK_eff_ or total NK were tested with a linear mixed model with the *lmer* function in the lme4 package with a logarithmic transformation to approximate normality by Shapiro testing. CD16 MFI was normalized applying an arcsinh (hyperbolic inverse sine) transformation with 150 cofactor; samples with missing data were removed. Both cellular count ratios and CD16 MFI were analyzed using the same Gaussian model with condition as the fixed effect and individual as a random effect, as previously described (30). To find statistically significant correlations between parameters, Spearman’s test was used. A p-value of ≤0.05 was considered significant.

## Results

### Clinical Features of Pediatric Patients With T1D Throughout the Study

Clinical data from control subjects and patients with T1D at each time-point are summarized in **Table 1**. All patients were positive for autoantibodies to GAD65, IA-2, and/or ZnT8. No statistically significant differences were found in age and BMI when compared between control subjects and patients at disease onset; as expected, they were found in terms of plasma C-peptide concentration. Differences at PR stage were found in BMI, HbA1c, insulin dose, C-peptide, and IDAA1c values when compared to recently diagnosed patients. These differences were not found 12M after the onset, time at which the plasma C-peptide concentration reached the minimum values. Non-remitter patients (8M) presented higher HbA1c and IDAA1c values than remitter patients, although they were still lower than those of newly diagnosed patients. Moreover, non-fasting C-peptide remained as low as at the onset of the disease.

**Table 1:** Clinical data for pediatric control subjects and patients with T1D at disease onset, PR, 8M (no PR), and 12M of progression.

	Control subjects (n = 17)	Patients at Disease onset (n = 17)	Patients at PR (n = 11)	Patients at 8M (no PR) (n = 6)	Patients at 12M (n = 6)
**Age (years)**	8.8 ± 3.3	8.7 ± 3.6	9 ± 4.1	8.5 ± 3	9.3 ± 3.6
**Sex (M/F)**	7/10	7/10	5/6	2/4	2/4
**BMI (kg/m^2^)**	18.3 ± 4.3	16.8 ± 2.5	17.7 ± 3.0^*^	17.2 ± 2.1	17 ± 1.7
**HbA1c (%)**	ND	11.4 ± 2.4	6.8 ± 0.6^**^	8.1 ± 0.7^*^/^xx^	7.6 ± 1
**HbA1c (mmol/mol)**	ND	101.3 ± 26.2	51.5 ± 6.4^**^	64.5 ± 7.4^*^/^xx^	59.2 ± 10.8
**Insulin dose (U/Kg/day)**	ND	0.7 ± 0.2	0.4 ± 0.1^**^	0.85 ± 0.1^***^	0.7 ± 0.1
**C-peptide (ng/ml)**	1.3 ± 0.4	0.3 ± 0.2^++++^	0.7 ± 0.5^++^	0.25 ± 0.1^+++^	0.1 ± 0.1^+++^
**IDAA1c (%)**	ND	14.3 ± 3	8.4 ± 0.5^**^	11.5 ± 1^*^/^xxx^	10.2 ± 1.4
**Autoantibodies IA-2A (+/−)**	ND	13/2	ND	ND	ND
**Autoantibodies GAD65A (+/−)**	ND	13/2	ND	ND	ND
**Autoantibodies ZnT8A (+/−)**	ND	11/6	ND	ND	ND
**High-risk HLA-DRB1 genotype**	ND	8/16	ND	ND	ND
**Moderate risk HLA-DRB1 genotype**	ND	7/16	ND	ND	ND
**Neutral HLA-DRB1 genotype**	ND	1/16	ND	ND	ND

Data are presented as mean ± SD; p-value calculated from Mann-Whitney test when comparing controls subjects and pediatric patients at the different time-points (^++^p ≤ 0.01, ^+++^p ≤ 0.001, and ^++++^p ≤ 0.0001) or from Wilcoxon test when comparing between patients at the different time-points (*p ≤ 0.05, **p ≤ 0.01, and ***p ≤ 0.001 for comparison with disease onset and ^xx^p ≤ 0.01 and ^xxx^p ≤ 0.001 for comparison with PR). PR, partial remission; M, male; F, female; BMI, body mass index; HbA1c, glycated hemoglobin; IDAA1c, insulin dose-adjusted HbA1c; IA-2A, anti-insulinoma antigen-2 autoantibody; GAD65A, anti-glutamic acid decarboxylase 65 autoantibody; ZnT8A, anti-zinc transporter 8 autoantibody; HLA, human leukocyte antigen; ND, not determined.

HLA typing of DRB1 alleles was determined in patients with T1D to determine genetic predisposition for the disease. As expected, 50% of the patients presented a high-risk HLA-DRB1 genotype and 44% of them were DRB1*03 or *04 positive, having a moderate risk for T1D. Only one patient was negative for DRB1*03 and *04 (neutral risk genotype) (**Table 1**).

### NK Cells and Their Effector, Regulatory, and Intermediate NK Subsets Are Altered at the Early Stages of T1D

**Figure 1:**
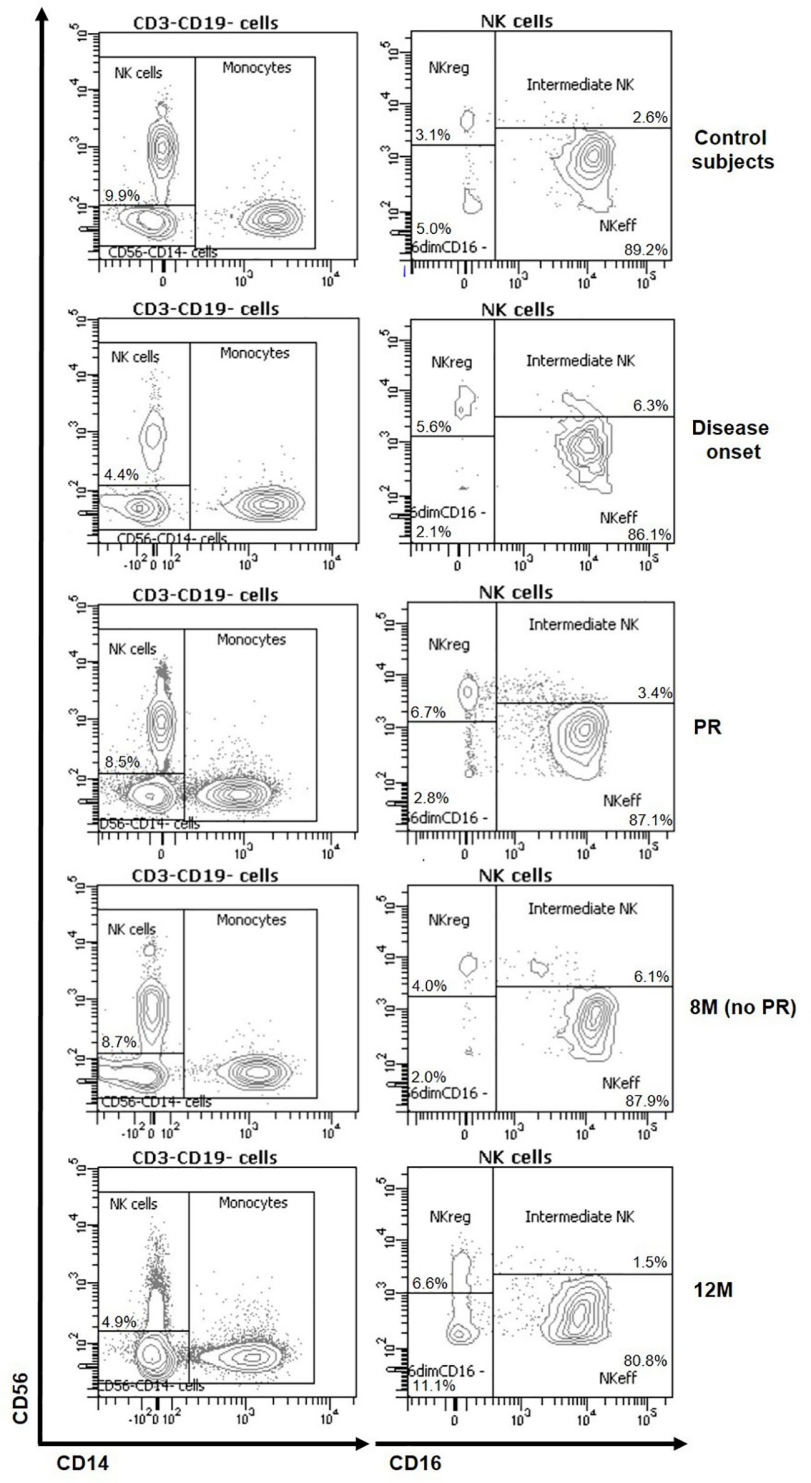
Representative plots of NK subsets changes at the initial stages of T1D. Representative staining for CD56 and CD14 on CD3^−^CD19^−^ cells and for CD56 and CD16 on total NK cells of control subjects (first row) and patients at disease onset (second row), at partial remission (PR, third row), 8 months without partial remission (8M no PR, fourth row) and at 12 months after onset (12M, fifth row).

To analyze the frequency and absolute counts of the four NK cell subsets in T1D patients at disease onset, PR, no PR (8M), and after 12M of progression, NK cells were detected as scatter-gated peripheral blood mononuclear cells (PBMCs), CD3^-^CD19^-^, CD56^+^, CD14^−^ and CD16^+ or −^ (**Supplementary Figure 1**). Representative plots for CD56 and CD14 on CD3^−^CD19^−^ cells and for CD56 and CD16 on total NK cells of the different groups are depicted in **Figure 1**.

**Figure 2:**
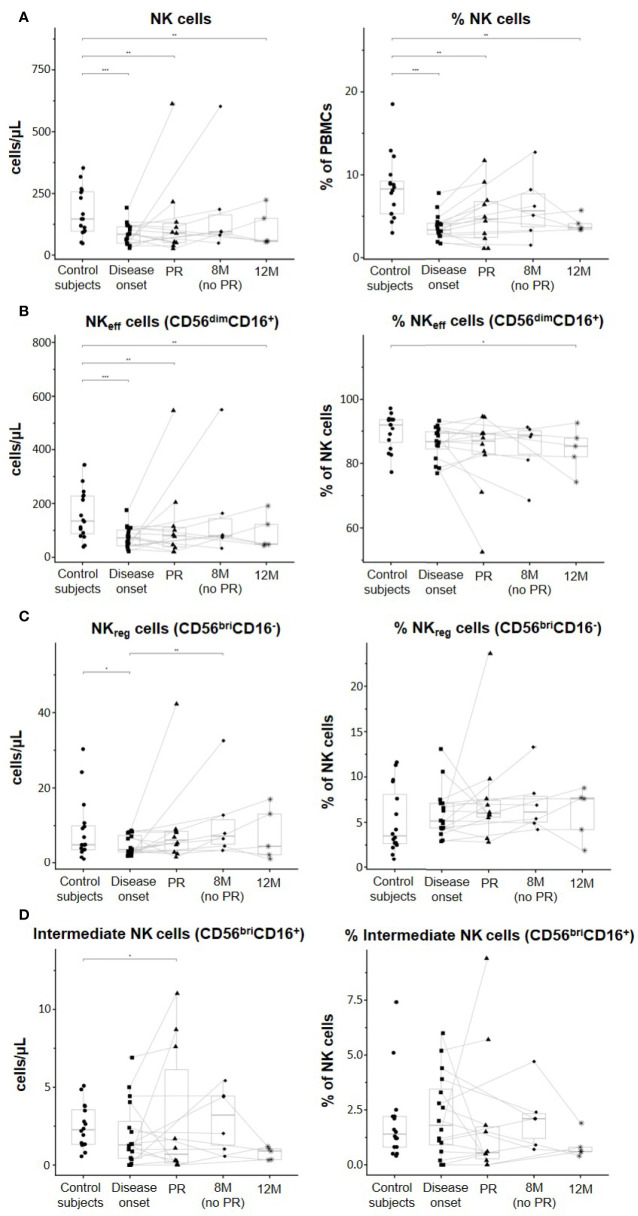
Peripheral blood NK subsets are quantitatively altered at the initial stages of T1D. Concentration (cells/µl, left panel) and percentages (%, right panel) of **(A)** total NK cells, **(B)** effector NK (NK_eff_) cells, **(C)** regulatory NK (NK_reg_) cells, and **(D)** intermediate NK cells. Dots represent control subjects, and patients are represented by squares at disease onset, triangles at partial remission (PR), rhombuses at 8 months without partial remission (8M no PR), and asterisks at 12 months after onset (12M). Lines link the same patient throughout the different time-points. Boxes indicate the median, first and third quartile range, and whiskers indicate the overall range without outliers of n ≥ 5 individuals per group. (*p ≤ 0.05, **p ≤ 0.01, and ***p ≤ 0.001, generalized linear mixed model).

**Figure 2** shows quantitative alterations of NK cell subsets in peripheral blood at all the described groups and time-points, as well as changes per patient during the year follow-up. As for total NK cells, percentages referring to total PBMCs were decreased in patients at disease onset (p ≤ 0.001), PR (p ≤ 0.01), 8M for non-remitter patients (p = 0.07), and 12M (p ≤ 0.01) when compared to control subjects. However, non-remitter patients showed a higher percentage of NK cells in comparison to patients at onset and patients undergoing PR, although non-significant. Their absolute counts also followed the same tendency, with lower counts of NK cells at disease onset (p ≤ 0.001), PR (p ≤ 0.01), and 12M (p ≤ 0.01) when compared to control subjects (**Figure 2A**). Referring to total NK cells, we also found diminished percentages of NK_eff_ cells in patients with T1D at all the different time-points when compared to control subjects, reaching a statistically significant decrease at 12M (p ≤ 0.05). Moreover, their absolute counts also decreased at disease onset (p ≤ 0.001), PR (p ≤ 0.01), and 12M (p ≤ 0.01) when compared to control subjects (**Figure 2B**). By contrast, NK_reg_ cells tended to increase in percentage in patients with T1D at all the time-points when compared to control subjects, although no statistically significant differences were observed. When compared to the disease onset, these cells also tended to present higher levels at the PR phase and 8M of disease progression (for non-remitter patients), reaching the highest level at 12M. Concerning absolute counts, patients at disease onset showed decreased levels when compared to control subjects (p ≤ 0.05), and NK_reg_ cells recovered at PR and 8M of disease progression, reaching in the last case a significant difference when compared to patients at T1D onset (p ≤ 0.01) (**Figure 2C**). Furthermore, we found that remitter patients and patients at 12M tended to diminish the percentage of intermediate NK cells, while non-remitter patients maintained or even increased their levels in comparison to the control subjects or patients at disease onset. Concerning the absolute counts, intermediate NK cells significantly decreased at the PR stage when compared to control subjects (p ≤ 0.05). At 12M, lower counts similar to those of the PR can be observed (**Figure 2D**).

**Figure 3:**
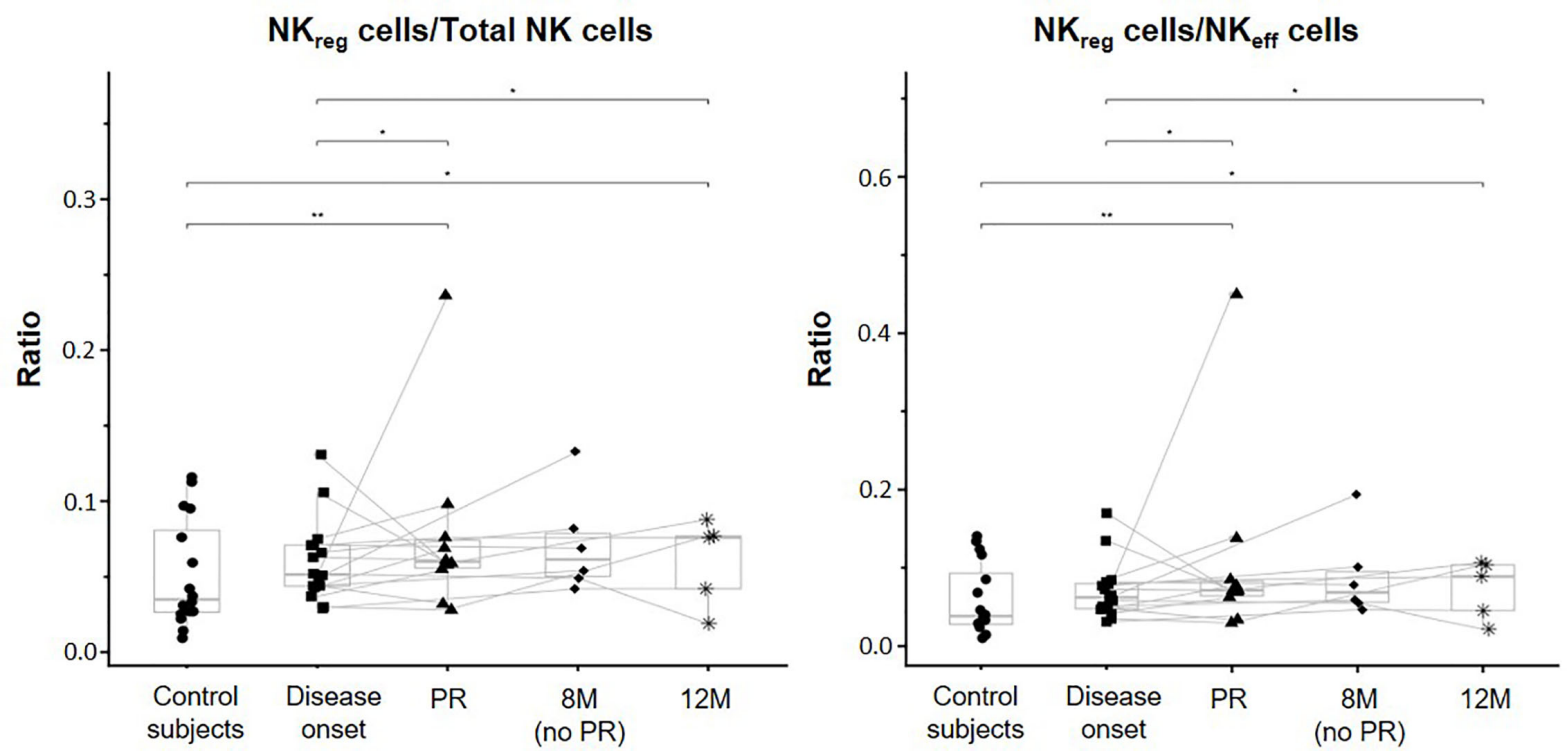
Changes in regulatory NK cells at initial stages of T1D. NK_reg_ cells/Total NK cells and NK_reg_ cells/NK_eff_ cells ratios were measured for each control subject (dots) and patient with T1DM at different time-points: disease onset (squares), partial remission (PR, triangles), 8 months without partial remission (8M no PR, rhombuses), and 12 months after onset (12M, asterisks). Lines link the same patient throughout the different time-points. Boxes indicate the median, first and third quartile range, and whiskers indicate the overall range without outliers of n ≥ 5 individuals per group. (*p ≤ 0.05 and **p ≤ 0.01, linear mixed model).

Although when looking at the data individually, heterogeneity can be found between patients in all the different NK cell subsets analyzed, the majority of them tended to increase the percentage and concentration of NK_reg_ at PR, 8M, and 12M when compared to T1D onset. In fact, the ratios between NK_reg_ cells and NK_eff_ cells and between NK_reg_ cells and total NK cells were both significantly higher at the PR phase in comparison to the ratios of control subjects (p ≤ 0.01) and disease onset (p ≤ 0.05). Also, the 12M time-point showed both higher ratios than control subjects (p ≤ 0.05) and T1D onset (p ≤ 0.05) (**Figure 3**).

When referring NK cell subsets to total PBMCs, percentages of NK_eff_ cells were significantly decreased in patients at disease onset (p ≤ 0.001), PR (p ≤ 0.01), and 12M (p ≤ 0.01) when compared to control subjects (**Supplementary Figure 2A**). Like total NK cells, these diminished percentages were not observed in non-remitter patients, who showed a higher percentage of NK_eff_ cells in comparison to the disease onset and patients undergoing PR, although non-significant. Regarding NK_reg_ cells, their percentages in peripheral blood significantly decreased at T1D onset in comparison to control subjects (p ≤ 0.05) and tended to recover again at the PR phase. In non-remitter patients, a significant recovery can be observed at 8M of disease progression when compared to the disease onset (p ≤ 0.01). Nevertheless, this increase might not be maintained over the months (**Supplementary Figure 2B**). Furthermore, we found that the percentage of intermediate NK cells tended to decrease at disease onset and at the PR phase, reaching in this last case a statistically significant reduction when compared to control subjects (p ≤ 0.05), but not at 8M of disease progression in the case of non-remitter patients (**Supplementary Figure 2C**). At 12M it can also be observed lower percentages when compared to control subjects.

### CD56dimCD16- NK Cell Subset Is Altered at 12 Months of T1D Progression

**Figure 4:**
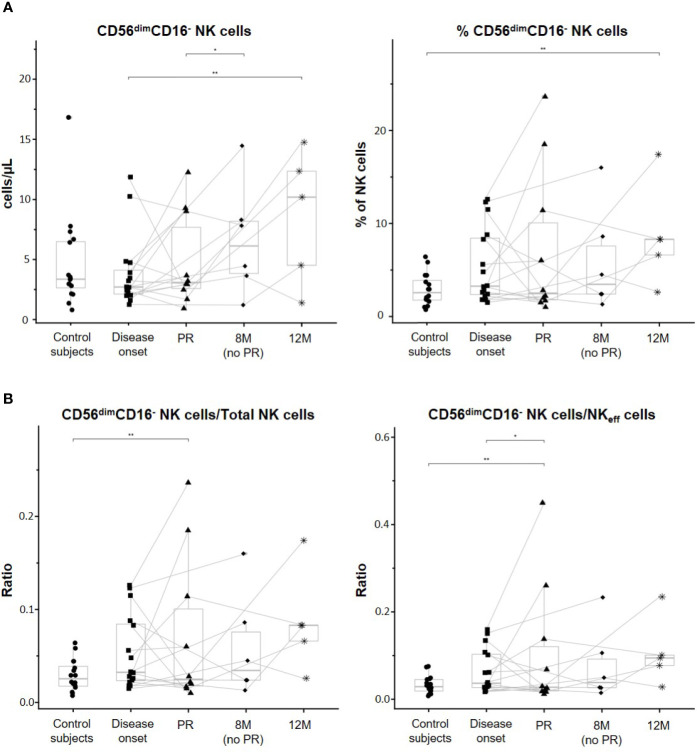
CD56^dim^CD16^-^ NK cell subset is altered along with the progression of T1D. **(A)** Concentration (cells/µl, left panel) and percentages (%, right panel) of total CD56^dim^CD16^−^ NK cells. **(B)** Ratios between CD56^dim^CD16^−^ NK cells and total NK cells (left panel) and between CD56^dim^CD16^−^ NK cells and effector NK (NK_eff_) cells (right panel). Dots represent control subjects, and patients are represented by squares at disease onset, triangles at partial remission (PR), rhombuses at 8 months without partial remission (8M no PR), and asterisks at 12 months after onset (12M). Lines link the same patient throughout the different time-points. Boxes indicate the median, first and third quartile range, and whiskers indicate the overall range without outliers of n ≥ 5 individuals per group. (*p ≤ 0.05 and **p ≤ 0.01, generalized linear mixed model and linear mixed model).

CD56^dim^CD16^−^ NK cells are a unique and not fully characterized subset of non-classical NK cells. First, albeit no differences in percentages were found between the control group and T1D onset, a trend to increase CD56^dim^CD16^−^ NK cells can be noted. While the percentages of these cells did not differ between patients at onset and remitters or non-remitters, a clear increase can be observed at 12M of disease progression when compared to the other time-points, and especially when compared to the control subjects (p ≤ 0.01). Regarding their concentration, CD56^dim^CD16^-^ NK cells significantly increased at 12M in comparison to patients at disease onset (p ≤ 0.01). Interestingly, a trend to expand this subset in peripheral blood with disease progression can be noted, and at 8M, non-remitter patients presented higher counts than remitter patients (p ≤ 0.05) (**Figure 4A**). Although when looking at the data individually heterogeneity can be found between patients regarding these cells, it is interesting to observe how they clearly increased in number at 12M in those patients that undergo the PR phase. By analyzing the ratios of CD56^dim^CD16^−^ NK cells/Total NK cells and CD56^dim^CD16^−^ NK cells/NK_eff_ cells, we obtained that patients at the PR phase presented both lower ratios than control subjects (p ≤ 0.01) and in the case of the CD56^dim^CD16^−^ NK cells/NK_eff_ cells ratio, than patients at disease onset (p ≤ 0.05). We also obtained higher ratios at 12M of disease progression than those of the other conditions, although not significant (**Figure 4B**). Altogether, these results indicate alterations in both concentration and percentage of the CD56^dim^CD16^−^ NK cell subset during the early progression of pediatric T1D.

When referring CD56^dim^CD16^−^ NK cells to total PBMCs, percentages of these cells were significantly increased at 12M of T1D progression when compared to the onset (p ≤ 0.01). Also, non-remitter patients showed a higher percentage of this subset in comparison to the remitter ones (p ≤ 0.05) (**Supplementary Figure 2D**).

### NK Cells and Their Subsets Do Not Correlate With Clinical Features at T1D Onset

**Figure 5:**
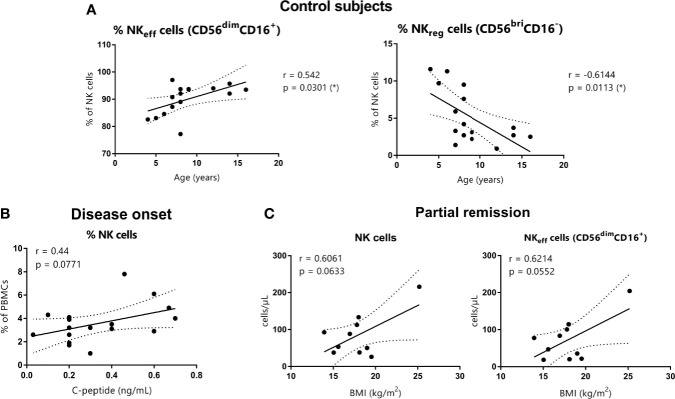
Correlations between NK cells and age, C-peptide, and BMI. **(A)** NK_eff_ cells and NK_reg_ cells percentages of control subjects are shown in correlation to age (years) (respectively, Spearman’s *r* = 0.542, *p* = 0.0301 and Spearman’s *r* = −0.6144, *p* = 0.0113). **(B)** NK cell percentages of patients at disease onset are shown in correlation to basal C-peptide (ng/ml) (Spearman’s *r* = 0.44, *p* = 0.0771). **(C)** NK cells and NK_eff_ cells absolute counts of patients at the PR phase are shown in correlation to BMI (kg/m2) (respectively, Spearman’s *r* = 0.6061, *p* = 0.0633 and Spearman’s *r* = 0.6214, *p* = 0.0552). (r and p-values by Spearman’s correlation analysis, *p ≤0.05).

In the control subjects group, a positive correlation between the percentage of NK_eff_ cells and age (Spearman’s *r* = 0.542, p = 0.0301) and a negative correlation between the percentage of NK_reg_ cells and age (Spearman’s *r* = −0.6144, p = 0.0113) were found (**Figure 5A**). However, these correlations were lost in T1D patients. Also, no significant correlations were found between total NK cells or their subsets and BMI, HbA1c, and C-peptide at T1D onset, neither in absolute counts nor in percentages. However, C-peptide tended to positively correlate with the percentage of total NK cells (% of PBMCs) (Spearman’s *r* = 0.44, p = 0.0771) (**Figure 5B**). At PR, absolute counts of both total NK cells and NK_eff_ cells tended to positively correlate with BMI values (respectively, Spearman’s *r* = 0.6061, p = 0.0633 and Spearman’s *r* = 0.6214, p = 0.0552) (**Figure 5C**).

### Modulation of CD16 Expression at Early Stages of T1D

**Figure 6:**
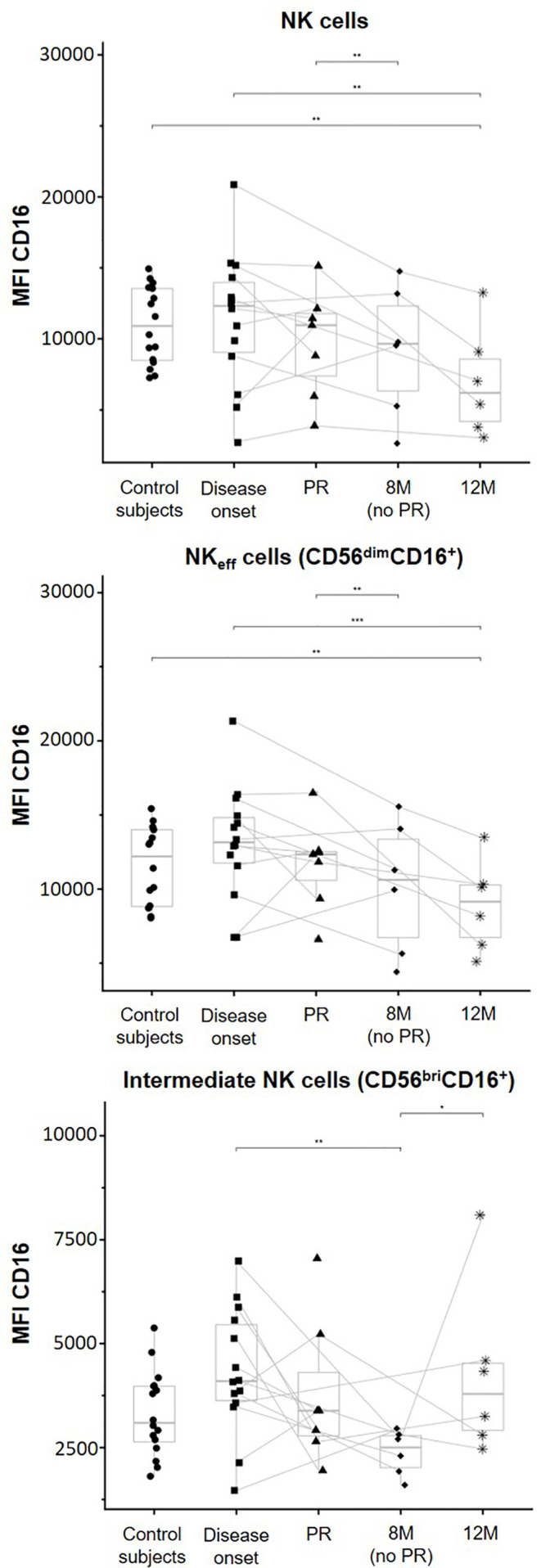
CD16 membrane expression is modulated depending on the phase of the disease. Median of fluorescence intensity (MFI) of CD16 surface expression on NK cells (upper graph), NK effector cells (NK_eff_ cells, middle graph), and intermediate NK cells (lower graph). Dots represent control subjects, and patients are represented by squares at disease onset, triangles at partial remission (PR), rhombuses at 8 months without partial remission (8M no PR), and asterisks at 12 months after onset (12M). Lines link the same patient throughout the different time-points. Boxes indicate the median, first and third quartile range, and whiskers indicate the overall range without outliers of n ≥ 5 individuals per group. (*p ≤ 0.05, **p ≤ 0.01, and ***p ≤ 0.001, linear mixed model).

To determine ADCC potential, CD16 expression levels in the NK cell membrane were assessed by flow cytometry. A tendency to a lower CD16 expression over time in patients with T1D can be observed on both total NK cells and NK_eff_ cells. Total NK cells of patients at 12M of disease progression expressed lower CD16 MFI levels than NK cells of control subjects (p ≤ 0.01) and patients at T1D onset (p ≤ 0.01). Furthermore, these cells of non-remitter patients exhibited a lower CD16 expression in comparison to those of remitter patients (p ≤ 0.01) (**Figure 6**, upper graph). CD16 expression was determined in the two positive subsets, NK_eff_ cells, and intermediate NK cells. The MFI of CD16 in NK_eff_ cells was similar to that of total NK cells. In the same way, NK_eff_ cells of patients at 12M of disease progression expressed lower CD16 MFI levels than NK_eff_ cells of control subjects (p ≤ 0.01) and patients at disease onset (p ≤ 0.001), and these cells of non-remitter patients also reduced the expression of CD16 in comparison to the remitter ones (p ≤ 0.01) (**Figure 6**, middle graph). Finally, in the intermediate NK cells, CD16 membrane expression tended to be higher at disease onset in comparison to control subjects. Following the same tendency as the NK_eff_ cells, CD16 also tended to be reduced at the PR phase; however, intermediate NK cells of non-remitter patients are marked by a reduction in the membrane expression of CD16 in comparison to all the other groups, especially to both patients at disease onset (p ≤ 0.01) and patients at 12M (p ≤ 0.05) (**Figure 6**, lower graph). Although heterogeneity can be found between remitter and non-remitter patients, we can observe that the majority of both of them tended to decrease CD16 expression at 12M of disease progression on total NK and NK_eff_ cells, while its expression is decreased on intermediate NK cells both at PR and 8M (more remarkably) in comparison to the disease onset.

## Discussion

Despite different strategies capable of stopping the autoimmune process and promote β-cell recovery have been developed in the last years, none of them has achieved total remission in humans. This failure could be partially explained by the lack of biomarkers that allow the proper stratification of patients, the identification of patients with a better glycemic prognosis, and the discovery of optimal checkpoints for immune interventions, e.g., the spontaneous PR phase. In this sense, alterations of immune cell subsets in peripheral blood, including NK cells, could be candidate biomarkers for disease staging and for understanding the immunoregulatory changes that take place at the early stages of the disease. Specifically, NK cells have been detected in the pancreas of both experimental models and patients with T1D, and specific subsets of these cells are related to an increased risk of developing the disease (31). The functional differences of NK cell subpopulations could explain their also reported dual behavior, one that promotes β-cell destruction and another that protects against the autoimmune attack (14, 15, 32).

Nowadays, recent advances in single-cell RNA sequencing analysis has revealed novel NK cell subsets in human peripheral blood, allowing their deep characterization and a better stratification of these cells than the classical cytometry-based approach (33, 34). Also, this technique provides new insights into NK cell biology (35). However, biomarkers for clinical use must be quickly and easily obtained. In the present study, we relied on a simple and fast multicolor flow cytometry assay to study four NK cell subsets through the first year of T1D progression in pediatric patients.

We report here that NK cells are diminished in patients with T1D during this follow-up. Moreover, we have observed crucial changes in the four NK cell subsets at the different studied time-points, which could reflect the immunoregulatory attempts that patients undergo during the PR phase. Also, membrane expression of CD16 in NK cells differed at the different disease stages, specifically between remitters and non-remitters and 1 year after diagnosis.

The variations found in NK cell subsets both in concentration and percentage reflect the dynamic changes of the innate immune system at the early stages of T1D, as recently described (20). Following previous studies, our results showed a decrease in the number and percentage of total NK cells and NK_eff_ cells at disease onset when compared to control subjects (18, 20, 21), while the absolute counts of PBMCs are similar between groups (data not shown). This reduction is sustained throughout the study follow-up, both at the PR phase and one year after T1D onset. The presence of NK cells in healthy human pancreatic islets evidences the innate capacity of these cells to migrate to the pancreas (36) as well as in response to inflammatory mediators (37). In this sense, the immunophenotyping of the insulitis in T1D patients revealed the presence of NK cells (38, 39). The reduction in peripheral NK cells at T1D onset is often correlated to an activated phenotype, and these alterations are no longer observed in individuals with long-standing disease (40). Because NK cells are among the first ones to migrate to the pancreas upon cytokine activation, we propose that the reduction in NK_eff_ cells we observed in the periphery could be explained by an active migration of these cells into the pancreas and draining nodes. Taking into account the NK data from experimental models (10, 11, 32, 41, 42), these results point to an active role of these cells in the disease pathogenesis: in the islets, they will exert a cytotoxic function against β-cells, thus contributing to disease development. In fact, NK cells are responsible for accelerated T1D in non-obese diabetic (NOD) mice (12). Additionally, in this model, pancreatic NK cells were found to exhibit higher levels of activation markers, a more marked mature phenotype and greater proliferation rates in comparison to other mouse strains, which supports a role for these cells in promoting β-cell destruction (32).

However, controversial results have been reported in humans. One study described in subjects from 6 months to 2 years after disease onset a decreased responsiveness of NK_eff_ cells to IL-2 and IL-15 stimulation and aberrant signaling through the activating natural killer group 2 member D (NKG2D) receptor (19), which could also explain the reduction in peripheral NK cell frequency. Given that the chronic exposure to ligands recognizing their activating receptors can result in NK cell hyporesponsiveness (43–45), we propose that the observed NK cell reduction after 1 year of follow-up could be explained by their loss of functionality. Indeed, another study demonstrated that the reduction in NK cell activation (including NK_eff_ and NK_reg_ cells) in patients with T1D occurs approximately 1 year after the onset of the disease and that the lower expression of NKG2D in these cells was not a genetic defect, suggesting an indirect modulatory effect (40).

Alternatively, NK cells regulate adaptive immunity by suppressing autoreactive T cell responses and proliferation and by killing over-stimulated lymphocytes, macrophages, or dendritic cells (46, 47). In this sense, the here reported follow-up of pediatric patients with T1D showed a tendency to increase NK_reg_ cells in blood during the first stages of T1D, especially the first months after the disease onset, when the PR occurs. That might reflect an attempt at immunoregulation during the chronic autoimmune attack at secondary lymphoid organs, as NK_reg_ cells can mold the autoreactive response through anti-inflammatory cytokines such as IL-10 (48). Actually, CD56^bright^ NK cells are the predominant subset in secondary lymphoid organs since they express CCR7 and CD62L (49). In other autoimmune diseases, such as multiple sclerosis, these CD56^bright^ NK cells exercise a regulatory role by suppressing self-reactive T cell responses and they expand after a successful response to immunotherapies (50). In NOD mice treated with Complete Freund’s Adjuvant, NK cells were the ones to suppress β-cell-specific T cells and to prevent diabetes (51). Nevertheless, the exhaustion of these cells over time might result in sustained macrophage and β-cell-specific T cell activation and enhanced proinflammatory responses, contributing in this way to the end of the PR stage.

Regarding intermediate NK cells, a transitional form between NK_reg_ and NK_eff_ cells with cytotoxic capacity, the observed reduction at the PR phase could be due to the immunomodulation of early stages. It has been described that T-cell derived cytokines and cell-cell contacts are needed for the acquisition of a functional CD16 and the transition of NK_reg_ cells into the intermediate NK subset (17, 49). Therefore, the immunoregulatory attempts during the PR phase may be dampening this conversion. Finally, CD56^dim^CD16^−^ NK cell subset increased through disease progression and non-remitter patients showed higher numbers of these cells than the remitter ones. In pediatric healthy subjects, this subset is more abundant in bone marrow than in peripheral blood (52), but it is not fully characterized in terms of function and ontogeny. However, antitumor cytotoxic activity and effector functions stronger than other NK subsets have been demonstrated (53, 54). Therefore, the increase in these cells during T1D progression could reflect their higher migration from bone marrow towards peripheral blood and possibly the pancreas, where they could exert their cytotoxic functions against β-cells. On the contrary, this phenomenon could be less frequent during the PR phase due to the lower peripheral counts found in this stage, contributing in this way to the immunoregulation. The source of this subset is controversial, and they can arise from either precursor NK cells (CD56^−^CD16^−^) that start to express the CD56 marker or NK_eff_ cells that lose membrane CD16 after they have been activated, thus becoming CD56^dim^CD16^−^ cells (24). Both mechanisms could contribute to the here reported progressive increase of these cells during the early stages of T1D.

In order to assess whether metabolism affects NK cell variations, several correlations were determined. No statistically significant correlations were observed between age, BMI, C-peptide, or HbA1c, and the different NK cell subsets of T1D patients in this study. As previously described (55), we also have detected statistically significant correlations in control subjects between the age and the percentage of NK_eff_ (positive correlation) and NK_reg_ cells (negative correlation). Although our pediatric cohort presents a wide range of ages, having found no correlations between this parameter and the different NK cell subsets in T1D reflects the immunological imbalance present in patients regardless of the age. While other studies showed a negative correlation between absolute counts of NK cells and the concentration of C-peptide (20), we observed that C-peptide tended to positively correlate with the percentage of total NK cells at disease onset. Moreover, during the PR phase, absolute counts of both total NK cells and NK_eff_ cells tended to positively correlate with higher BMI values. These results might reflect that better glycemic control and the removal of glucose toxicity could impact the migration of NK cells during the remission period, thus increasing in blood and contributing less to the autoimmune attack. Interestingly, it has been shown that patients with diabetic ketoacidosis at diagnosis display a lower frequency of NK_reg_ cells (21). Rodacki et al. found a tendency of NK cells to negatively correlate with HbA1c in recently diagnosed but not in long-standing patients (40), suggesting that NK cell reduction is not solely caused by the loss of glucose control. Nevertheless, further research is needed to confirm the impact of glucose metabolism on NK cell subsets.

Further in this study, we determined the expression of CD16 because it can hint at the NK cell functional state as this receptor is involved in ADCC. As mentioned before, CD16 on NK cells suffers a shedding process after cell activation and its expression is reduced after multiple cell-to-cell interactions, leading NK cells to a state of exhaustion  (24, 56). This effect could be the reason for the observed reduction in CD16 expression on total NK cells and NK_eff_ cells in patients with T1D after diagnosis. Despite it is difficult to establish if this reduction is caused by NK cell degranulation and CD16 shedding or by the downregulation of CD16 membrane expression, these results could support the progression to a state of hyporesponsiveness after 1 year of disease evolution. Concerning intermediate NK cells, this subset increases the expression of CD16 during the differentiation process from NK_reg_ to NK_eff_ cells  (57). In our study, these cells showed a tendency to increase CD16 membrane expression at disease onset in comparison to controls. This might reflect a strong cytotoxic profile and the ongoing differentiation into NK_eff_ cells. Although non-remitter patients at 8M had similar intermediate NK cell percentages as the disease onset, we found a remarkable decrease in CD16 membrane expression when compared both to the T1D onset and 12M. That could be explained by a higher number of NK_reg_ cells differentiating into intermediate NK cells in non-remitter patients (which would still express lower levels of CD16) since no immunomodulatory effect is stopping this process. This transition could also be prompted by the necessity to resupply the exhausted NK_eff_ cell pool. At 12 months, the recovery of CD16 membrane expression could reflect an enhanced cytotoxic activity of these cells and their conversion into the NK_eff_ subset  (17).

We are well aware of the limitations of the present study. The main ones are the lack of functional experiments that makes it purely descriptive and the small sample size that lowers the statistical power. Also, another major weak point of our study population is the wide range of ages (from 4 to 18) and the high variability that this entails regarding the physiological changes that the subjects undergo (e.g., puberty, among others). Despite this, longitudinal data were analyzed with a generalized linear mixed model, and age was excluded from the final model due to a lack of significant effects. Moreover, the p-value of the NK subsets determines statistical differences and the obtained results are in accordance with those of previous studies (20–22). As mentioned before, functional studies are required in order to elucidate potential mechanisms of action of NK cell subsets and to deepen more in the characterization of reliable and reproducible biomarkers for the PR phase. The higher levels of NK_reg_ cells found during the first months after the disease onset or the diminished concentration of CD56^dim^CD16^−^ and intermediate NK cells in remitter patients could be potential candidate biomarkers reflecting the immunoregulatory attempts at the early stages of T1D. However, this work should be considered as a pilot study, and further examination in larger cohorts is needed to validate our observations. Furthermore, the analysis of the targeted organ and other cell subsets would be beneficial to clarify the exact role of each of the NK subsets and to link peripheral NK cell variations with the pancreatic events. Nevertheless, the strength of this study is the reported longitudinal picture of NK cell subsets during T1D progression, including the analysis at the PR phase, which enables us to explore the underlying pathophysiology and understand the immunoregulation during the early stages.

Overall, the here reported results show clear alterations of peripheral NK cell subsets during the progression of T1D, which can reflect both their involvement in the autoimmune attack and their contribution to the immunoregulatory attempts during the PR stage.

## Data Availability Statement

The original contributions presented in the study are included in the article/**Supplementary Material**. Further inquiries can be directed to the corresponding author.

## Ethics Statement

The studies involving human participants were reviewed and approved by Committee on the Ethics of Research of the Germans Trias i Pujol Research Institute and Hospital (HUGTiP) and Hospital Parc Taulí. Written informed consent to participate in this study was provided by the participants’ legal guardian/next of kin.

## Author Contributions

LG-M, DP-B, AV, SR-F, AT-S, and MV-P designed the experiments. FV, MM, JP, RC, and JB selected the patients and obtained the samples. LG-M, DP-B, AV, R-MA, and SR-F performed the experiments; LG-M, DP-B, AV, SR-F, and MV-P analyzed the data and wrote the manuscript. All authors contributed to the article and approved the submitted version.

## Funding

This study has been funded by Instituto de Salud Carlos III through the project PI18/00436 (Co-funded by European Regional Development Fund ‘A way to make Europe’), and by DiabetesCero Foundation.

## Conflict of Interest

MV-P holds a patent that relates to immunotherapy for T1D and is co-founder of Ahead Therapeutics S.L. SR-F is part-time employed at Ahead Therapeutics S.L.

The remaining authors declare that the research was conducted in the absence of any commercial or financial relationships that could be construed as a potential conflict of interest.

## Abbreviations

ADCC, antibody-dependent cell cytotoxicity; BMI, Body Mass Index; HbA1c, Glycated Hemoglobin; IDAA1c, Insulin Dose-Adjusted HbA1c; M, Months; MFI, Median Fluorescence Intensity; NK, Natural Killer; NK_eff_, Effector Natural Killer; NKG2D, Natural Killer Group 2 member D; NK_reg_, Regulatory Natural Killer; NOD, Non-obese Diabetic; PBMCs, Peripheral Blood Mononuclear Cells; PR, Partial Remission; T1D, Type 1 Diabetes.
