# Corrigendum: NK cell subsets changes in partial remission and early stages of pediatric type 1 diabetes

**DOI:** 10.3389/fimmu.2024.1439246

**Published:** 2024-06-05

**Authors:** Laia Gomez-Muñoz, David Perna-Barrull, Adrian Villalba, Silvia Rodriguez-Fernandez, Rosa-Maria Ampudia, Aina Teniente-Serra, Federico Vazquez, Marta Murillo, Jacobo Perez, Raquel Corripio, Joan Bel, Marta Vives-Pi

**Affiliations:** ^1^ Immunology Service, Germans Trias i Pujol Research Institute, Autonomous University of Barcelona, Badalona, Spain; ^2^ Endocrinology Service, Germans Trias i Pujol University Hospital, Badalona, Spain; ^3^ Pediatrics Service, Germans Trias i Pujol University Hospital, Badalona, Spain; ^4^ Department of Pediatric Endocrine, Parc Tauli Hospital Universitari, Institut d’Investigació i Innovació Parc Taulí I3PT, Universitat Autonoma de Barcelona, Sabadell, Spain

**Keywords:** NK cell subpopulations, type 1 diabetes, partial remission, pediatrics, autoimmunity

In the published article, there was an error in the **Funding** statement. The **Funding** statement was displayed as “This work has been funded by the Spanish Government (FIS PI18/00436) co-financed with the European Regional Development funds (FEDER), and by DiabetesCero Foundation.”. The correct **Funding** statement appears below.

**FUNDING**

This study has been funded by Instituto de Salud Carlos III through the project PI18/00436 (Co-funded by European Regional Development Fund ‘A way to make Europe’), and by DiabetesCero Foundation.

The authors apologize for this error and state that this does not change the scientific conclusions of the article in any way. The original article has been updated.

