# Supplementary material for: NK Cell Subsets Changes in Partial Remission and Early Stages of Pediatric Type 1 Diabetes
Source: Front Immunol. 2021 Jan 25;11:611522. doi: 10.3389/fimmu.2020.611522 (PMC7869615; doi:10.3389/fimmu.2020.611522)
Supplement: Supplementary file 1 [file Table_1.docx]

*SUPPLEMENTARY MATERIAL*

SUPPLEMENTARY FIGURES


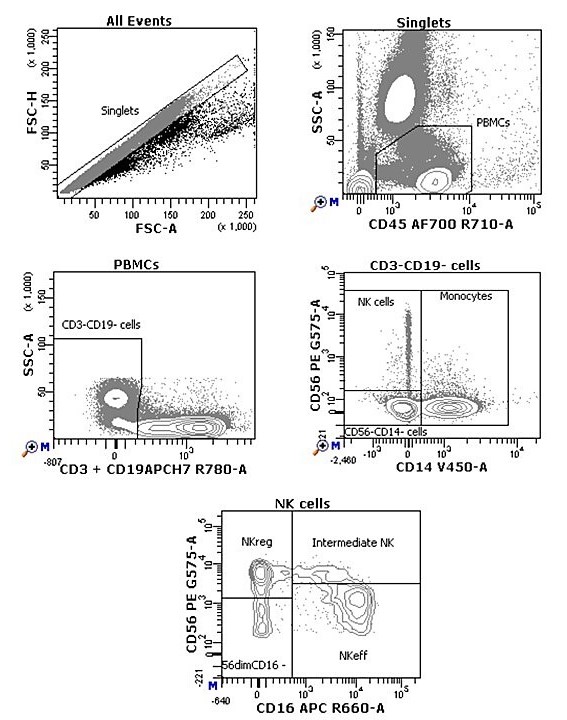


Supplementary Figure 1. Representative gating strategy for NK cells panel. Gating strategy used to analyze the percentages and absolute counts of the four NK cell subsets. Cells were first gated for singlets, PBMCs (CD45+ cells), and CD3-CD19- cells. With the use of CD56 and CD14, cells were divided into negative lineage, NK cells (CD56+CD14-) and monocytes (CD14+). NK cells were further divided into four subsets: regulatory NK cells (NKreg, CD56briCD16-), effector NK cells (NKeff, CD56dimCD16+), intermediate NK cells (CD56briCD16+), and CD56dimCD16- cells.


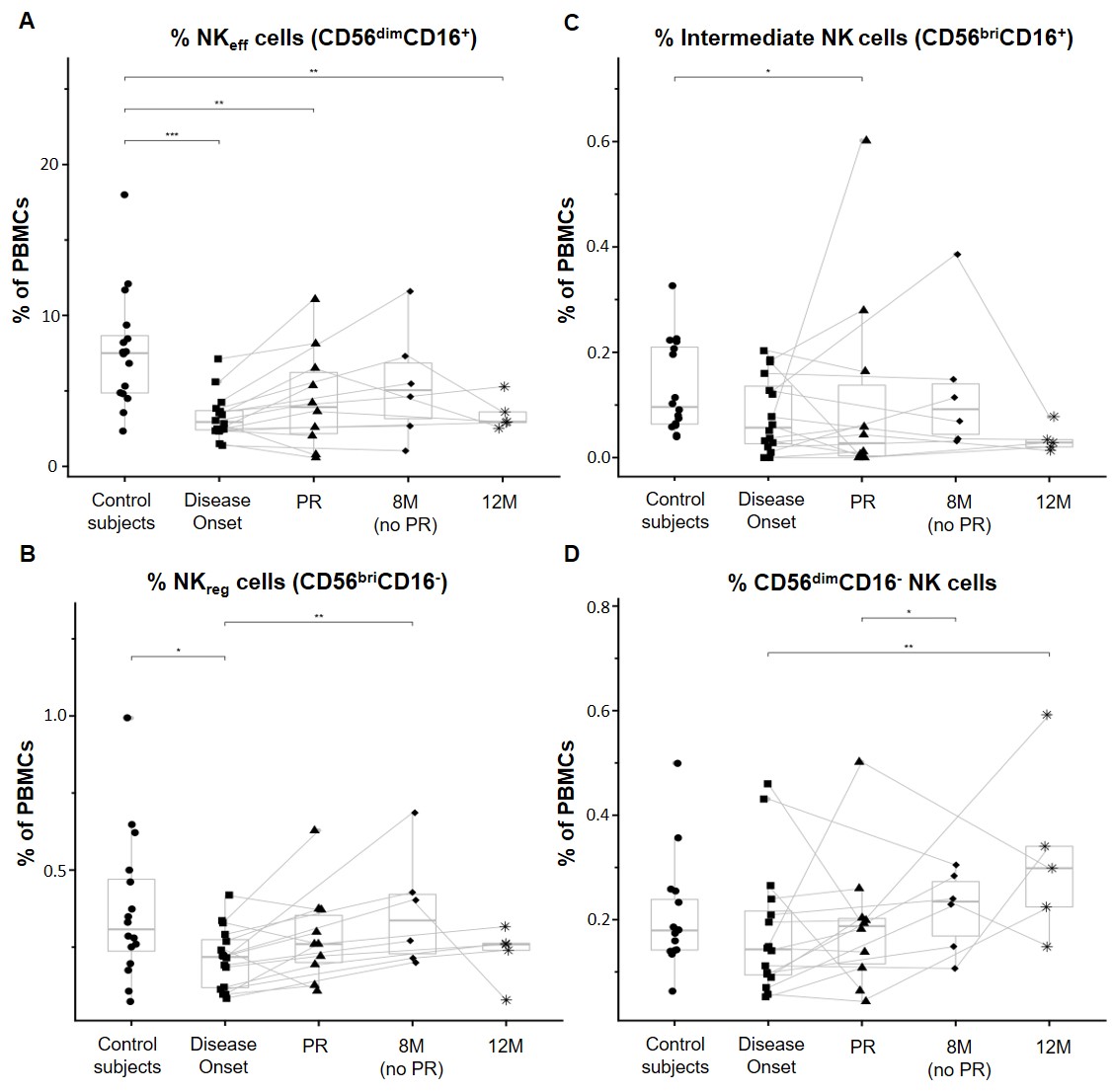


Supplementary Figure 2. Percentages of peripheral blood NK subsets are quantitatively altered at the initial stages of T1D. Percentages (% of PBMCs) of (A) effector NK (NKeff) cells, (B) regulatory NK (NKreg) cells, (C) intermediate NK cells, and (D) CD56dimCD16- NK cells. Dots represent control subjects, and patients are represented by squares at disease onset, triangles at partial remission (PR), rhombuses at 8 months without partial remission (8M no PR), and asterisks at 12 months after onset (12M). Lines link the same patient throughout the different time-points. Boxes indicate the median, first and third quartile range, and whiskers indicate the overall range without outliers of n ≥ 5 individuals per group. (*p ≤0.05, **p ≤0.01 and ***p ≤0.001, generalized linear mixed model).
